# Epidemiological, Poisoning Characteristics and Treatment Outcomes of Patients Admitted to the National Poisoning Control Centre at Karachi, Pakistan: A Six Month Analysis

**DOI:** 10.7759/cureus.6229

**Published:** 2019-11-25

**Authors:** Anum Amir, Farhan Haleem, Ghulam Mahesar, Rukhsana Abdul Sattar, Talha Qureshi, Jabbar Ghufran Syed, M Ali Khan

**Affiliations:** 1 Internal Medicine, Civil Hospital, Karachi, PAK; 2 Gastroenterology, Jinnah Postgraduate Medical Centre, Karachi, PAK; 3 Internal Medicine, Jinnah Postgraduate Medical Centre, Karachi, PAK; 4 General Surgery, Jinnah Postgraduate Medical Centre, Karachi, PAK

**Keywords:** poisoning, organophosphate poisoning, epidemiology, outcome, karachi, pakistan

## Abstract

Introduction: Any substance if taken in enough quantity can be defined as a poison provided it causes physiological or anatomical harm. It can range from food products to therapeutic medications to toxins and chemicals. Animals, plants, and insects also produce toxins, which are poisonous. While any route of ingestion is dangerous, most poisons are either taken by mouth or inhaled. Rarely intravenous access as in the case of heroin/opoids overdose is seen as well.

Poisoning whether deliberate or otherwise is a growing problem of the modern world. Young people are disproportionally affected by it. Mostly household products such as insecticides, bleach, acid, etc. are used. Harmful ingestion of prescription meds, recreational drugs, psychiatric medicines, and opoids has been on the rise in recent times. This is one of the major sources of poisoning these days.

Data with respect to Sindh and Pakistan is scarce. As the largest referral center in the country, Jinnah Postgraduate Medical Centre sees its fair share of poisoning cases. Here we evaluate the trends and increasing burden of poisoning cases seen at this center.

Aims: To evaluate the epidemiological, poisoning characteristics and treatment outcomes of patients admitted to the National Poisoning Control Centre (NPCC) at Karachi, Pakistan.

Materials and methods: This is a retrospective study, held from July 1^st^ to December 31^st^ 2018. Data were recorded from all patients admitted to the NPCC after complete medico-legal work up.

Results: A total of 2546 patients were inducted into the study. The mean age of presentation was 26.57 ± 11.82 years. Nearly 80% of patients were aged 40 years or younger. Both genders were equally affected and most cases were referred from within the city. Organophosphates (OPs) were the most frequent (46.11%) cause of poisoning seen. Overall mortality was 3.61%.

Conclusion: The burden of poisoning cases has risen sharply. Mostly young adults and teenagers are affected without gender bias. Mortality is high considering the young population involved.

## Introduction

Poison can be defined as any substance that causes harm when ingested or breathed in or injected or absorbed through the skin. Even normally nonpoisonous substances such as vitamins, caffeine, and therapeutic drugs when taken excessively can become poisonous. Most cases of poisoning presenting to the hospitals are deliberate.

The number of poisoning cases has steadily increased over the last two decades in Sindh. Most of these cases are due to oranganophosphate (OP) ingestion [1]. Other causes include alcoholism, psychiatric medications, acid ingestion, beta blocker overdose, alkali ingestion, fabric softener ingestion, and narcotics. Most poisoning centers also treat cases of animal poisoning such as jellyfish sting, snake and spider bites, and scorpion stings.

The complex socio-economic, psychological, and other aspects of poisoning are beyond the scope of this article. Almost all of the cases are of suicidal intent [2] and undergo medico-legal examination and registration at the ER, given appropriate resuscitative measures, and then admitted. Such sensitive information is kept off limits to most personnel.

Because of the above-mentioned reasons the data are scarce on poisoning trends in Sindh especially with respect to the largest referral center of the province for poison control, i.e. National Poisoning Control Centre (NPCC) at Karachi. Here we look back at the cases that were admitted in the last six months of 2018 to evaluate the trends and outcomes, in light of the data that was made available to us.

## Materials and methods

Study design

Retrospective.

Setting

National Poisoning Control Centre, Medical Unit I (Ward 5), Jinnah Postgraduate Medical Centre, Karachi, Pakistan.

Induction criteria and duration

All patients admitted to the NPCC from July 2018 to December 2018, with a valid medico-legal number were inducted into the study.

Information disclosure

All patients admitted to the NPCC undergo extensive examination by a medico-legal officer; a report is filed on the officer’s part with its assigned registration number. Thus, the case becomes a part of the justice/legal system. Most of the information such as treatment regimen given, suicidal or homicidal nature of the poisoning and other illnesses could not be accessed for this study and were off limits.

Patient confidentiality was made certain. All patients were referred to by their given numbers. The variables available have been analyzed in this study. 

Sample technique

Nonprobability, consecutive sampling.

Data analysis

Statistical package for social sciences (SPSS) version 21 was used to analyze the data.

## Results

Total admissions

A total of 2546 patients were admitted from July 1 to December 31. This constituted approximately 10% of all admission to the unit and about 3% of all referrals from the ED within the six month study period. 

Demographics

Demographics are summarized in Table 1. Most patients were young with a mean age of 26.57 ± 11.82 years. Largest number of patients was seen in the 20-40 year age group. There was no statistical significance between the genders of the patients. Referrals were made mostly from the within the city or the province..

**Table 1:** Demographics and epidemiological characteristics.

	N=2546
Gender	
Male	1311 (51.45%)
Female	1235 (48.55%)
Location	
Karachi	2357 (92.61%)
Sindh	151 (5.89%)
Baluchistan	33 (1.33%)
Punjab	5 (0.15%)
Age groups	
≤12 years	20 (0.78%)
13-19 years	755 (29.69%)
20-40 years	1527 (59.93%)
41-59 years	156 (6.12%)
60≥ years	94 (3.69%)
Overall age (mean)	26.57±11.82 years

Poisoning types

Organophosphate (OP) ingestion was hands down the most common type of poisoning seen. Off-label products and rat killer poison were the most common OP ingested. Types of poisoning are summarized in Table 2. 

**Table 2:** Different types of poisoning analyzed during the study.

	N=2546
Organophosphates	1174 (46.11%)
Off-label products	458 (17.98%)
Rat killer	282 (11.07%)
Insecticides	194 (7.61%)
Typhoon	190 (7.46%)
Phenyle	50 (1.96%)
Snake bite(s)	231 (9.07%)
Over the counter pills	225 (8.80%)
Bleach ingestion	194 (7.61%)
Sleeping pills	86 (3.37%)
Heroin/opoids overdose	58 (2.27%)
Insect bite(s)	53 (2.07%)
Kerosene/Diesel ingestion	46 (1.86%)
Acid ingestion	32 (1.25%)
Scorpion sting	21 (0.82%)
Alcohol	12 (0.47%)
Methanol	10 (0.39%)
Ethanol	2 (0.07%)
Blackstone	12 (0.47%)
Copper sulfate	9 (0.35%)
Indeterminate poisoning	393 (15.43%)

Mortality and subgroup analysis

Overall mortality was low. Not surprisingly greatest numbers of death were attributed to OP poisoning. Similarly OP ingestion (and mortality) was also associated with longer hospital stay. Mortality and length of stay are summarized in Table 3. Mortality for specific poisons is shown in Table 4.

**Table 3:** Overall mortality, discharge frequency, and length of stay at the hospital.

	N=2546	Length of stay (mean in days)
Discharged	2331 (91.51%)	1.87±1.59
Expired	92 (3.61%)	2.48±3.40
Left against medical advise	123 (4.88%)	1.46±0.95

**Table 4:** Absolute and relative mortality rates for specific poisons.

	Mortality N (%)	Relative mortality within the poisoning type group (%)
Organophosphates (all)	70 (2.7%)	5.96%
Off-label products	38 (1.49%)	8.29%
Typhoon	16 (0.62%)	8.42%
Rat killer	11 (0.43%)	3.90%
Insecticide	5 (0.19%)	2.57%
Blackstone (paraphenylene-diamine)	8 (0.31%)	66.66%
Methanol overdose	5 (0.19%)	41.66%
Snake bite	3 (0.11%)	1.29%
Acid ingestion	2 (0.07%)	6.25%
Kerosene ingestion	2 (0.07%)	4.34%
Heroin overdose	2 (0.07%)	3.44%
Overall	92 (3.61%)	

## Discussion

The number of poisoning cases has sharply risen over the last three decades. Comparing data from the same unit shows an increase from a single patient every two days [3] to more than 20 patients per day; this study shows a more than 40-fold increase. These numbers reflect the rising population, easier access to a hospital, improved intercity transportation, and rapid urbanization of Karachi.

Patients were mostly referred from within the city or the province, but few were referred from other provinces as well. The next registered poisoning center is approximately 600 km away and this explains referrals from areas that are in between. This usually means late presentation with more severe symptoms. However, no correlation between outcomes and referrals outside the province could be made. 

Men and women were almost equally affected with a median age was 23 years; this has remained unchanged for the last three decades [4]. Almost 80% patients were under the age of 40; teenagers constituted almost a third of patients. This is consistent with previous studies and evidence suggests most of it is deliberate self harm [5].

Any substance can be used as a poison, but OP ingestion accounted for most cases. This is in part due to their easy availability, large volume (liquid or solid) that is available to ingest, and an understanding of its toxic potential by the patient. It is the opinion of the author that it is the last aspect which makes them the go to poison for most patients.

While the percentage of OP poisoning was consistent with Pakistani and international data, there was a changing trend in the type of OP poisoning. Most of our patients took an off-label product. While it is declared on the “label” that this is an OP, there are neither regulations nor instructions mentioned on the labels. These are multipurpose and are used for many a reason. These are also the cheapest and perhaps the weakest concentrate of OP available.

Other OP compounds such as rat killers, insecticides made up the remainder of cases. Second most common cause of admission was snake bites (along with scorpion and insect bites); also the most common cause of unintentional harm. Most of the areas adjoining Karachi are flat lands with wild shrubs and some lakes and marshes, along with most of the interior province of Sindh. These make an ideal habitat for snakes and other insects ergo the high number of cases. Unfortunately most of the snakes, insects, and scorpions are taxonomically unmarked and remain unidentified.

Incidence of alcohol poisoning was low. This masks the fact that there are outbreaks on public holidays secondary to local moon shining businesses. These are associated with severe methanol overdosing and an exceptionally high mortality rate. Just because one such outbreak did not occur during the study period, one should not play down the impact of alcohol poisoning on society. An entire study arc should be separately held on alcoholism. 

Indeterminate cases include false reporting of intake, ingesting harmless substances, and reporting them as poison or falsely reporting symptoms associated with other substance abuse. There was no mortality associated with indeterminate cases suggesting benign nature of the cases at least acutely. Pill ingestion (pain killers, analgesics, sleeping pills) make up about 12% of all cases. Due to their benign nature and massive doses required for poisoning, no deaths were reported in either group.

Of note here is that acute liver failure secondary to acetaminophen ingestion is not treated at the poisoning unit and was not a part of this study. Bleach, kerosene, and acid ingestion initially had relatively good outcomes; however, whether these patients developed gastrointestinal complications such as bleeding, perforation, stricture, and fibrosis could not be followed up. Blackstone is a dyeing agent and was associated with the highest mortality (discussed below).

Overall mortality rate for OP ingestion and poisoning in general was similar to previous international data [6-7]. Two thirds of the patients with Blackstone poisoning expired. Blackstone is a dyeing agent commonly used for hair. It contains an intensely toxic compound known as paraphenylene-diamine (PPD). This can cause multiorgan damage such as angioneurotic edema (especially laryngeal edema), rhabdomyolysis, and acute tubular necrosis with renal failure. There is almost always a need for tracheotomy tube place or laryngeal intubation. Even early and aggressive management is associated with poor outcomes.

Methanol overdose had the second highest mortality. As mentioned earlier outbreaks can occur on joyous occasions and our data are not representative of that fact. Patients usually present with intoxication, blindness, encephalopathy, brain damage and/or ischemia, electrolyte and micronutrient disturbances, renal failure and eventually death. In most cases even at presentation the prognosis was guarded as multiorgan failure had ensued.

Mortality for acid and kerosene ingestion was in all cases associated with an extremely high volume of intake and development of aspiration pneumonia. Deaths due to heroin overdosing occurred in addicts who had poor nutritional status and could not be worked up for other comorbidities. Length of stay for those discharged is reasonably short.

There is some distortion in length of stay (mean) for patients who expired. Most international data suggest a median of seven days. In our study it is three days. This can be attributed to the fact some patients present late, when poisoning has effected multiple systems. This leads to an early mortality and therefore reducing the mean.

Shortcomings

This study does not take into account the complex socio-economic factors influencing poisoning cases. Neither does this study take into account the various psychological aspects that affect a patient who is willing to do self harm. Patients were not followed up.

## Conclusions

Poisoning cases have increased at an alarming rate. They usually affect young adults and teenagers. Most of the poisons are easily accessible and available in most of the households or shops especially OPs. Mortality is high considering the age groups involved. A new comprehensive effort from social and medical sectors is required to reduce its negative impact.

## References

[1] Jan A, Khan MJ, Humayun Khan MT (2016). Poisons implicated in homicidal, suicidal and accidental cases in North-West Pakistan. J Ayub Med Coll Abbottabad.

[2] Shaikh S, Khaskheli MS, Meraj M (2018). Effect of organophasphate poisoning among patients reporting at a tertiary healthcare facility of Sindh Pakistan. Pak J Med Sci.

[3] Jamil H (1990). Acute poisoning--a review of 1900 cases. J Pak Med Assoc.

[4] Khan NU, Khan UR, Feroze A (2016). Trends of acute poisoning: 22 years experience from a tertiary care hospital in Karachi, Pakistan. J Pak Med Assoc.

[5] Shahid M, Hyder AA (2008). Deliberate self-harm and suicide: a review from Pakistan. Int J Inj Contr Saf Promot.

[6] Hussain AM, Sultan ST (2005). Organophosphorus insecticide poisoning: management in surgical intensive care unit. J Coll Physicians Surg Pak.

[7] Aardema H, Meertens JH, Ligtenberg JJ (2008). Organophosphorus pesticide poisoning: cases and developments. Neth J Med.

